# Exploring online health information seeking and sharing among older adults: a mini-review about acceptance, potentials, and barriers

**DOI:** 10.3389/fdgth.2024.1336430

**Published:** 2024-01-19

**Authors:** Yves Bachofner, Alexander Seifert, Samin Sepahniya, Carlo Fabian

**Affiliations:** ^1^ Institute for Social Work and Health, School of Social Work, University of Applied Sciences and Arts Northwestern Switzerland, Olten, Switzerland; ^2^ Institute for Integration and Participation, School of Social Work, University of Applied Sciences and Arts Northwestern Switzerland, Olten, Switzerland

**Keywords:** eHealth, health literacy, older adults, online health, health seeking

## Abstract

Online health information seeking (OHIS) is understood by health care, health promotion, and disease prevention experts as a resource for healthy aging. It is particularly relevant for older adults since this population can benefit significantly from the accessibility and convenience of online health platforms and health information. Nevertheless, empirical findings regarding the acceptance, potentials, and barriers of OHIS among older adults are limited. This mini-review aims to explore the level of acceptance of OHIS, including passive reading of information and active interactions with peers, among the older population. Furthermore, it examines the potentials and barriers associated with such practices. The findings ultimately emphasize the evolving landscape of internet health information exploration among older adults and the potential advantages and challenges that may arise, especially in the context of active interactions with peers.

## 1 Introduction

In an increasingly digital era, the use of electronic health (eHealth) resources has become a central component of modern health care systems, health promotion, and disease prevention (1). Among these resources, the internet in particular has provided a new means of searching for health information, and it offers unprecedented potential for older adults to assume a more pronounced partnership role in managing their health and decision-making about health care (2, 3). Furthermore, by engaging in health-related exploration via social media platforms, older adults can fulfill their need for health information and access social and emotional support through peer group interactions (4), as observed during the COVID-19 pandemic (5).

Using eHealth to seek health-related information and sharing such information with others is particularly relevant for older adults, especially those aged 60 years or older. Indeed, this population group can benefit significantly from the accessibility and convenience of eHealth platforms and health-related information. eHealth presents a possible solution to overcome the barriers faced by older adults when they attempt to access timely, effective, and adequate health information regarding health care, health promotion, and disease prevention in old age (6). At the same time, because of the complexity of the health status of seniors and the risk of information overload, older adults may encounter significant hurdles in online health information seeking (OHIS). These challenges include selecting from diverse information sources, formulating precise health queries, and evaluating misinformation (7). The basic requirement of OHIS for older people is that they have access to the internet (8, 9). Although access to the internet among older adults is not always the case, there is emerging evidence that this first digital divide in access has been closed (10). Nevertheless, the COVID-19 pandemic exposed a worldwide digital gap between younger and older generations, which indicates that older adults are also today not as familiar with the internet (11).

When older people confront age-related health issues, their interactions with online health information and digital channels influence their decision-making. Research has shown that the internet plays a vital role in facilitating distance-based interactions within the peer-group health sector, and its importance predated the COVID-19 pandemic (12, 13). Additionally, while eHealth applications have potential benefits for the aging population, there are also inherent risks related to existing and newly emerging social inequalities. The digital inequality within the older age population (8) and the disparity between older individuals who heavily use the internet and those who are not digitally literate have effects on existing health inequalities in old age that could increase or persist in the future (14).

In light of this context, this mini-review aims to answer two central questions: What is the level of acceptance among the older population regarding OHIS, specifically in relation to both the passive reading of information and active interaction with peers? And what are the potentials and barriers associated with passive and active OHIS? Addressing these questions can help to define gaps in the current research.

## 2 Methods of the mini-review

**Figure 1:**
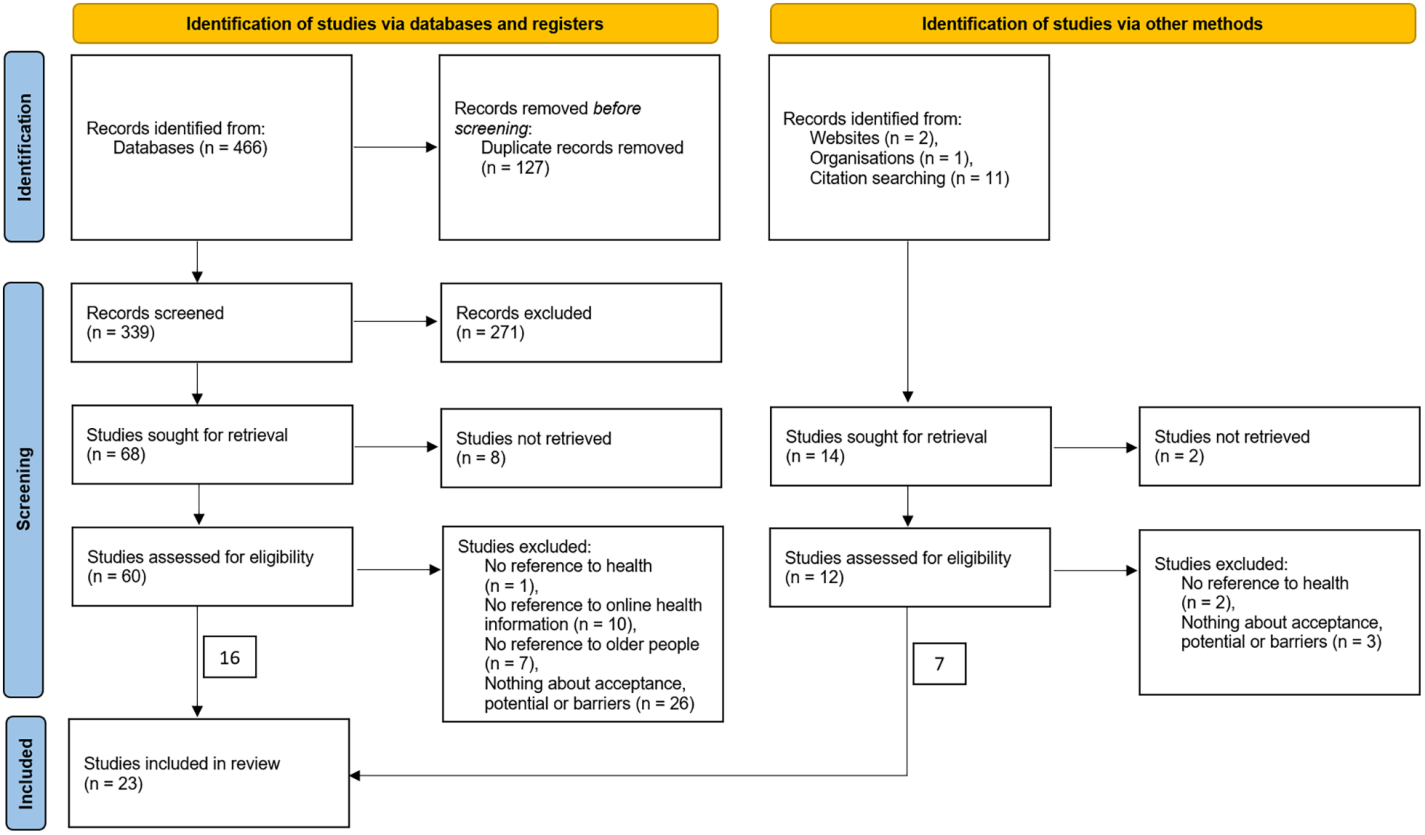
Flowchart of the literature screening process.

In this research, we conducted a systematic literature search of seven databases in May 2023, adopting a PRISMA-style approach (15): Sociological Abstracts; Ovid, including PsychInfo, Psyndex, and Eric; WISO database on economics and social sciences; Web of Science; Cinahl; Google Scholar; and Swisscovery, the Swiss library service platform. We used the research questions to develop the search strings. The search terms were framed *a priori* using Boolean logic. The following main keywords were used in the search strings: (1) acceptance, (2) use, (3) need, (4) barrier, (5) older people, (6) eHealth, and (7) OHIS. After removing duplicates, 339 articles were screened. The screening process included 14 additional articles that were identified by other methods (see Figure 1). After screening the abstracts, we excluded 271 articles. Two members of the research team then independently reviewed the full texts of the remaining 68 articles for eligibility. The exclusion criteria and flowchart of the selection process are presented in Figure 1. To meet the inclusion criteria, the articles had to focus on online health information, target older people (aged 60 years or older), be published in English or German between January 2015 and May 2023, and explore OHIS acceptance, potential, or barriers. After screening the full-text articles, 23 articles were included (Figure 1). Supplementary Table S1 provides an overview of the selected studies with a systematization regarding the key areas of acceptance, potential, and barriers, as well as the form of information (seeking or sharing).

## 3 Results

### 3.1 Acceptance of OHIS by older adults

#### 3.1.1 General acceptance of OHIS

The acceptance of online health information among older adults is a multifaceted phenomenon encompassing various aspects of digital engagement for health-related purposes. A common theme in previous studies is that older adults are increasingly utilizing the internet to inform themselves about their health (8, 14, 16, 17). Furthermore, the prevalent use of popular social media platforms (e.g., Facebook, Twitter) for this purpose underscores the digital adaptation of the older population to well-known online platforms (14). Similarly, findings have revealed that a significant portion of older individuals use the internet as a means of accessing valuable health-related information (8, 16). For example, in one study, over 70% of older internet users searched for health-related information online (17). The study identified three types of information seeking: primarily for oneself (43.6%), for others (11.6%), or for both one's own and others’ needs (15.3%).

Yet, in contrast, other studies have indicated lower levels (around 10%) of internet use among the older population to seek health-related information (18) or specifically search for information about cancer (19). Notably, however, this group used other channels (e.g., health care professionals, social networks, newspapers, television, radio) more frequently.

This digital transition is not uniform across demographic groups (20, 21). Some individuals within the older population, especially those aged 65 years or older, are digitally savvy and use the internet intensively; others, mostly aged 80 years or older, are less familiar with and skilled at using the internet, and they therefore favor more traditional means of seeking health information (6, 22). The varying uptake of internet-based health services across European countries also reflects different levels of adoption and access (20, 21). Further correlations can be established between internet use and factors such as educational status and cognitive function in the older population (21). There are differences in specific modes of access as well—for instance, in one study, fewer participants searched for health information on a stationary computer compared to on a mobile device, such as a smartphone (18).

Crucially, the effective use of the internet as a health information resource is contingent upon several factors. Perceived usefulness, explorative technology behavior, and health-related knowledge are pivotal in driving OHIS among the older population (23). Additionally, eHealth literacy, including in relation to OHIS (24), and perceived usefulness are significant factors in determining frequency of use, which reinforces the notion that informed and motivated individuals are more likely to engage effectively with digital health platforms (21).

#### 3.1.2 Older adults’ acceptance of active OHIS through peer interactions

The specific patterns within health-related online peer interactions among older adults have only been partially explored in current research. For example, participation in web-based support groups has been observed in only a small proportion of studies, and attention to engagement as a contributor to online health diaries and blogs has been even rarer (14). However, the use of mobile phones has been noted as a means of connecting with other patients and exchanging information (18). This observation underscores the diversity of communication channels available to older adults for participating in health-related peer discourse.

Research has also examined the dynamic landscape of online health social support groups (OSHSGs) as avenues for health-related peer interactions, such as the sharing of health-related information with peers, and emphasized the increasing propensity to disclose personal health information within these virtual communities. This shift has been driven by the accessibility, affordability, and fast proliferation of social media and OSHSGs (25). Regarding motivations for using OSHSGs, it has been noted that the need for informational support outweighs the desire for nurturant support, which underscores the importance of knowledge acquisition and exchange (25). Nevertheless, the current findings suggest that the active use of OHIS to share health-related information with peers is currently limited in its acceptance among older adults.

### 3.2 The potential of passive and active OHIS among older adults

#### 3.2.1 Individual level

The potential of OHIS is that it presents a promising avenue to enhance the well-being of older individuals. In this context, the transformative influence of such information seeking has been evidenced in several studies (8, 9, 26, 27). Digital health offerings, including OHIS, have been promoted for their effectiveness, particularly in regard to prevention. These interventions aim to induce positive behavioral changes and cultivate personal responsibility among users (26).

A recent systematic literature review has addressed the transformative impact of eHealth interventions on older individuals. The findings reveal how these interventions bolster health literacy skills and empower older adults to identify trustworthy health resources, make informed choices, and elevate their overall quality of life by adeptly navigating health information available on digital platforms (9). Moreover, the diversity of online health information resources has been recognized for its potential to immensely benefit older adults (8).

The advantages derived from eHealth interventions, particularly in accessing health information, are especially notable for individuals with heightened health needs, such as those with chronic health conditions (23, 27). Additionally, individuals with higher health needs not only attribute greater value to eHealth-related management but also actively seek out new ways to use the technology (23). This proactive approach has increased the use of OHIS among this technologically savvy subgroup of older adults.

In essence, the potential of eHealth for older individuals lies in its capacity to empower them with information, promote informed decision-making, and cultivate a proactive stance toward health management (28). In one study concerning online peer support groups for prostate cancer, users viewed participation positively, which significantly impacted their decision-making (29). As the digital health landscape continues to evolve, these individuals stand to benefit substantially from the wealth of resources and interventions that are accessible online (28). Such a proactive stance can facilitate heightened utilization of OHIS (28).

Notably, OHIS facilitated by peer interactions has the potential to amplify health outcomes and cultivate health-promoting behaviors within the older population, as highlighted in a previous comprehensive review (30).

#### 3.2.2 Society level

The use of technology can facilitate more participation, partnerships, empowerment, and equity in a range of settings, provided that suitable conditions, such as equitable access to technology and sufficient usage competencies, are established (28). The expansion of internet-based health care, including the provision of online health information, has valuable potential for effectively managing and preventing chronic diseases, particularly among older adults (31).

Addressing the digital divide and enhancing access to health information through research efforts can be integral to reducing health disparities (20). The intention behind digital support for health-promoting behavioral changes, such as increasing physical activity and healthier eating, is to effectively mitigate risk factors, such as obesity, prior to engaging health care systems (26). Additionally, since people seek health information not only for themselves but also on behalf of others, they can influence the behavioral changes or health-related decisions of other people (10).

A recent review of eHealth literacy intervention programs (9) tailored to older adults has underscored two key societal benefits: improved health status and improved overall well-being. These benefits can be primarily attributed to heightened medical decision-making and problem-solving abilities in older individuals. Furthermore, the broader effects of improved health literacy and decision-making skills may include decreased emergency room utilization and a subsequent reduction in medical costs.

### 3.3 Barriers to OHIS for older adults

In general, the use of OHIS requires competent usage of the internet. However, such competency cannot be assumed for all older persons. Even today, there is still a digital divide, with very old persons (aged 80+ years) often rarely using the internet (16). Furthermore, prevalent age-related limitations (e.g., hearing and vision loss, memory deficits, reduced fine motor control) have been reported as significant obstacles to OHIS (6). Internet use has been associated with various socio-economic factors, including educational attainment, cognitive function, and the possession of internet-enabled devices (21).

Although the internet can supply valuable health information, it can exacerbate inequity. For instance, differences in internet skill and health literacy levels among the older population mean that those with few skills are not able to take advantage of OHIS (30). Likewise, a major barrier is a lack of skills related to searching for information (6, 18). Individuals who find it challenging to effectively browse the internet for relevant resources may struggle to access them (16). Moreover, limited experience and infrequent engagement in eHealth practices have been identified as hindering factors (6, 10). Users with less exposure to OHIS may not be as enthusiastic about practicing it, and past negative experiences can discourage active participation in OHIS and foster reluctance to trust online health resources (6).

Another critical obstacle is insufficient health literacy (7–10, 21, 23). Inadequate comprehension can prevent people from effectively interpreting and utilizing digital health-related resources. Language barriers and cultural beliefs about health and illness significantly impact eHealth literacy levels and the ways in which individuals assess and navigate digital health resources (32). Other barriers for older adults include safety concerns (6, 16), distrust, and disbelief in the effectiveness of OHIS (6).

Certain demographic variables can also affect the use of online health information by older adults. For example, the probability of using online health information is significantly less for individuals who have a lower monthly household income, live in a rural area, or work in agriculture (18). Additionally, less use of OHIS has been predicted for individuals with lower education levels (14, 18). Research has found that gender was a factor affecting the use of online health information, as women were almost three times more likely than men to use OHIS (14). Still, there is mixed evidence regarding age and gender as predictors of OHIS. A recent study found no significant correlation between age and OHIS despite using a wide age range in the sample, and gender was not deemed a significant factor either, except in relation to seeking information through peers (10).

## 4. Discussion

The growing use of OHIS among older adults represents a positive aspect of digital health care, health promotion, and disease prevention. As older adults are increasingly seeking health information on the internet, it has become apparent that digital platforms, including popular social media sites and online platforms, can serve as valuable health education and peer interaction resources for this population.

This mini-review has shown that older adults are increasingly using the internet to inform themselves about their health. Still, current rates of OHIS are rather low, which indicates that there is still a high utilization potential among the older population (17, 21). Today, older generations possess a higher level of technological proficiency compared to their predecessors, which is in line with the trend toward greater use of OHIS reported in the literature.

Additionally, it is crucial to weigh potential variations in use across demographic groups. Factors such as age, socioeconomic situation, marital status, cognitive ability, and method of access impact the extent to which older adults engage with online health information (14, 18, 21). These variances emphasize the need for tailored digital literacy policies and programs to ensure equitable access and use.

The online health-related interactions of older adults have introduced novel opportunities for information exchange, emotional sustenance, and collaborative undertakings. However, the motivations for online peer interactions within health support communities reveal a complex interplay between informational and emotional needs (25). Research has only marginally examined OHIS specifically through peer interactions among older adults. Considering the potential and impact of face-to-face peer support, it is vital to explore online peer-to-peer support and health-related exchanges as well. These insights can inform the development of platforms that satisfy a range of user needs and ultimately enhance the quality of online health discourse. At the same time, the possible risks—such as inaccurate information, lack of expertise, privacy concerns, overreliance on online sources, and vulnerability through openness—should always be considered and reduced (33).

In a society where health information is becoming increasingly digitized and prevalent, digital health literacy is arguably essential for older adults. Many research areas lack reliable and varied data on digital health literacy and the necessity of online health-related interactions among older adults. However, the digital divide and associated health disparities are steadily gaining importance. Consequently, the success of regional and national initiatives to improve OHIS depends on whether they address the specific needs of the older population regarding technology use and health literacy. Additionally, it is clear that improvement efforts at the regional and national levels are less effective when a significant portion of the population is excluded due to insufficient digital information literacy (32). This exclusion neglects their specific needs and interests and undermines community-building efforts.

### 4.1 Future directions

Future research should continue to explore the evolving landscape of OHIS among older adults. Evaluating the effectiveness of interventions and staying attuned to the changing role of technology in health care can inform evidence-based strategies for improving access and outcomes. Area-wide studies could also reveal regional differences that may guide the creation of more tailored platforms. Policy efforts should focus on addressing the digital divide, supporting digital literacy initiatives, and promoting the development of user-friendly online health resources.

This review has also demonstrated that, aside from the passive retrieval of information on the internet, the active exchange of health information between peers is a highly promising practice but has been only minimally discussed in the literature for the target group of older persons.

In summary, the findings of this mini-review illustrate the changing nature and potential benefits of OHIS among older adults. It has also clarified the barriers that must be addressed. Future research should further investigate the development and evaluation of interventions aimed at enhancing digital health literacy, resolving disparities, and improving online health information access for older adults.
