# Supplementary material for: Exploring online health information seeking and sharing among older adults: a mini-review about acceptance, potentials, and barriers
Source: Front Digit Health. 2024 Jan 19;6:1336430. doi: 10.3389/fdgth.2024.1336430 (PMC10834747; doi:10.3389/fdgth.2024.1336430)
Supplement: Supplementary file 1 [file Table1.docx]

Supplementary Material S1

Exploring Online Health Information Seeking and Sharing Among Older Adults: A Mini-Review about Acceptance, Potentials, and Barriers

Table S1: Selected studies

| Authors &Year | Title | Recruitment country | Type of Study | Accep-tance | Potential | Barrier | OHIS | OHIS & sharing |
| --- | --- | --- | --- | --- | --- | --- | --- | --- |
| Brainin & Neter (2023) | Refined analysis of older eHealth users from an agency perspective: Quantitative telephone interview study | Israel | Quantitative study |  | X | X | X |  |
| Choi (2020) | Factors associated with eHealth use among community dwelling older adults | South Korea | Quantitative secondary data analysis | X | X | X | X |  |
| Cornejo Müller et al. (2020) | Digital Divide – Soziale Unterschiede in der Nutzung digitaler Gesundheitsangebote |  | Literature analysis |  | X |  | X |  |
| Crouch & Gordon (2019) | Prevalence and factors influencing use of internet and electronic health resources by middle-aged and older adults in a US health plan population: Cross-sectional survey study | USA | Quantitative cross-sectional study | X | X |  | X |  |
| Hargittai et al. (2019) | From internet access to internet skills: digital inequality among older adults | USA | Quantitative study | X |  |  | X |  |
| Huber et al. (2018) | Face-to-face vs. online peer support groups for prostate cancer: A cross-sectional comparison study | Germany | Quantitative cross-sectional comparison study |  | X |  | X | X |
| Leung (2017) | Cancer-related information seeking and scanning behaviors among older Chinese adults: Examining the roles of fatalistic beliefs and fear | Hong Kong | Quantitative cross-sectional study | X |  |  | X |  |
| Merkel & Hess (2020) | The use of internet-based health and care services by elderly people in Europe and the importance of the country context: Multilevel study | European countries | Quantitative secondary data analysis | X |  |  | X |  |
| Norgaard et al. (2015) | The e-health literacy framework: A conceptual framework for characterizing e-health users and their interaction with e-health systems | Denmark & Great Britain | Qualitative concept mapping to generate a comprehensive and grounded model | X |  |  | X |  |
| Oh & Lim (2021) | Patient-provider communication and online health information seeking among a sample of US older adults | USA | Quantitative secondary data analysis | X |  |  | X |  |
| Reinwand et al. | Who follows eHealth interventions as recommended? A study of participants’ personal characteristics from the experimental arm of a randomized controlled trial | Netherlands | Quantitative randomized controlled trial |  | X |  | X |  |
| Rockmann et al. (2019) | Gesundheitsbezogene IT-Nutzung im Altersübergang – Ursachen und Auswirkungen individueller Differenzen | Germany & USA | Quantitative study | X | X | X | X |  |
| Seifert et al. (2020) | Digitale Senioren 2020. Nutzung von Informations- und Kommunikationstechnologien (IKT) durch Menschen ab 65 Jahren in der Schweiz | Switzerland | Quantitative cross-sectional study | X |  | X | X |  |
| Sharma & Khadka (2019) | Role of empowerment and sense of community on online social health support group | USA | Quantitative study | X |  |  |  | X |
| Shi et al. (2023) | In the digital age: A systematic literature review of the e-health literacy and influencing factors among Chinese older adults |  | Systematic literature review |  |  | X | X |  |
| Stark et al. (2023) | Zukunftstrends und Einsatzmöglichkeiten digitaler Technologien in der settingbezogenen Prävention und Gesundheitsförderung – eine Delphi-Befragung | Germany | Delphi survey |  | X |  | X |  |
| Stefanicka-Wojtas & Kurpas (2022) | eHealth and mHealth in Chronic Diseases—Identification of Barriers, Existing Solutions, and Promoters Based on a Survey of EU Stakeholders Involved in Regions4PerMed (H2020) | European countries | Qualitative survey |  | X |  | X |  |
| Tennant et al. (2015) | eHealth literacy and web 2.0 health information seeking behaviors among baby boomers and older adults. | USA | Quantitative cross-sectional study | X |  | X | X | X |
| Welch et al. (2016) | Interactive social media interventions to promote health equity: An overview of reviews. |  | Review |  | X | X | X | X |
| Wilson et al. (2021) | Barriers and facilitators to the use of e-health by older adults: a scoping review |  | Scoping review | X |  | X | X |  |
| Zhang et al. (2019) | Factors Affecting ICT Use in Health Communication among the Older Population in Jiangsu, China | China | Quantitative cross-sectional study | X |  | X | X | X |
| Zhao et al. (2022) | Online Health Information Seeking Behaviors Among Older Adults: Systematic Scoping Review |  | Scoping Review |  |  | X | X |  |
| Zolbin et al. (2022) | Health literacy, health literacy interventions and decision-making: a systematic literature review |  | Literature review |  | X | X | X |  |
